# Ciliated neurons lining the central canal sense both fluid movement and pH through ASIC3

**DOI:** 10.1038/ncomms10002

**Published:** 2016-01-08

**Authors:** Elham Jalalvand, Brita Robertson, Peter Wallén, Sten Grillner

**Affiliations:** 1 The Nobel Institute for Neurophysiology, Department of Neuroscience, Karolinska Institutet, Stockholm SE-171 77, Sweden

## Abstract

Cerebrospinal fluid-contacting (CSF-c) cells are found in all vertebrates but their function has remained elusive. We recently identified one type of laterally projecting CSF-c cell in lamprey spinal cord with neuronal properties that expresses GABA and somatostatin. We show here that these CSF-c neurons respond to both mechanical stimulation and to lowered pH. These effects are most likely mediated by ASIC3-channels, since APETx2, a specific antagonist of ASIC3, blocks them both. Furthermore, lowering of pH as well as application of somatostatin will reduce the locomotor burst rate. The somatostatin receptor antagonist counteracts the effects of both a decrease in pH and of somatostatin. Lateral bending movement imposed on the spinal cord, as would occur during natural swimming, activates CSF-c neurons. Taken together, we show that CSF-c neurons act both as mechanoreceptors and as chemoreceptors through ASIC3 channels, and their action may protect against pH-changes resulting from excessive neuronal activity.

## Abstract (web summary)

Cerebrospinal fluid-contacting (CSF-c) cells line the central canal of the vertebrate spinal cord yet their function remains unknown. Here, Javaland *et al*. show that CSF-c neurons in the lamprey respond to mechanical stimulation and lowered pH, effects likely mediated by ASIC3-channels.

## Graphical abstract

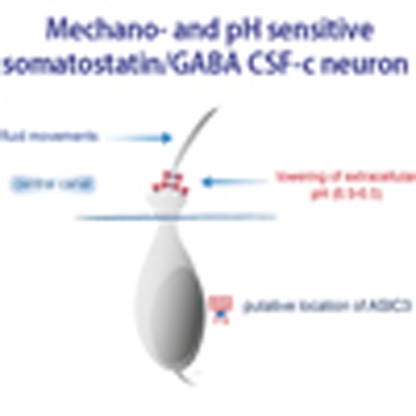

The central canal of the spinal cord is a conserved structure present in all vertebrates but with an unknown function. The width of this structure is miniscule, in the range of 10–20 μm, and it contains the Reissner´s fibre, a protein strand extending from the brainstem level to the caudal spinal cord. In the wall of the central canal there are cerebrospinal fluid-contacting (CSF-c) cells, the function of which has remained an enigma over more than a century^1^,^2^,^3^,^4^,^5^. Ideas regarding their function have varied from signalling fluid movements within the central canal to sensing the chemical composition of the cerebrospinal fluid^5^. Laterally projecting CSF-c cells in the lamprey spinal cord extend processes into the grey matter, and even as far as the lateral margin, where they form a plexus around the stretch-sensitive dendrites of stretch receptor neurons^3^,^6^,^7^. These CSF-c cells are of two types: (1) one with neuronal properties that displays action potentials, receives synaptic input (GABA- and glutamate-mediated), and expresses GABA and somatostatin; and (2) another with glia-like properties that displays no active currents or expression of neurotransmitters^3^. Optogenetic activation of GABAergic CSF-c neurons in larval zebrafish influences the locomotor network^8^, by slowing ongoing locomotor activity^9^, showing that these neurons provide input to the spinal locomotor central pattern generator^8^.

Our goal here is to resolve the functional role of the laterally projecting CSF-c neurons by performing patch clamp recordings while the cells are subjected to micro-jets of fluid or decreases in extracellular pH. The CSF-c neurons responded to both mechanical stimuli and lowering of pH, effects that were abolished by specific blockade of the acid-sensing ion channel, ASIC3. Furthermore, we show that both somatostatin and lowering of pH have a depressing effect on the fictive locomotor burst rate and that this effect was blocked by a somatostatin receptor antagonist. These results suggest that the network effects of lowering of the pH are mediated by somatostatin expressing CSF-c neurons, through an activation of ASIC3.

## Results

### CSF-c neurons are sensitive to fluid movements

**Figure 1:**
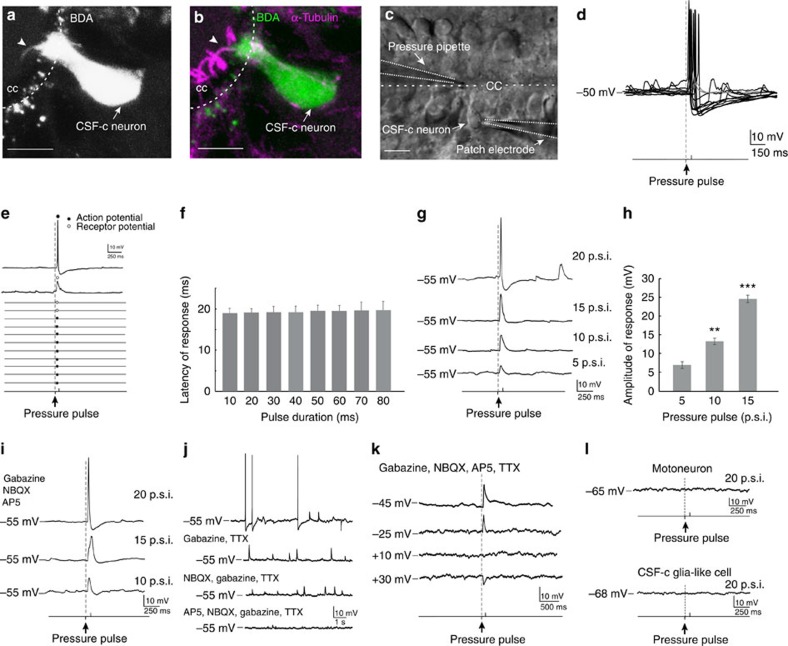
CSF-c neurons with cilia are sensitive to fluid movement. (**a**,**b**) A CSF-c neuron retrogradely labelled following BDA injection in the lateral margin. The cilium (arrowheads) is α-tubulin immunoreactive. (**c**) *In vitro* preparation with the central canal lumen exposed. A CSF-c neuron was patched and a Ringer-filled pressure pipette was placed close to its bulb-like ending. (**d**) A short (80 ms) fluid-pulse elicited receptor- or action potential responses. (**e**) The neuron responded with action- or receptor potentials at short and constant latency to all stimuli (20 p.s.i., 80 ms). (**f**) The response latency was around 20 ms irrespective of the duration of the fluid pulse (10–80 ms; means±s.e.m.; *n*=10). (**g**) Fluid-pulses of 5–15 p.s.i. elicited subthreshold receptor potentials of increasing amplitude, while a 20 p.s.i. pulse triggered an action potential response. (**h**) The mean receptor potential amplitude increased for all cells tested (*n*=6) in response to increasing fluid pulse magnitude. Means±s.e.m.; Student's t-test: **P<0.01 and ***P<0.001, significant difference compared with value at 5 p.s.i. (**i**) The response to fluid movement remains after application of GABA and glutamate receptor antagonists, gabazine (20 μM) and NBQX (40 μM)/AP5 (100 μM), respectively. (**j**) A CSF-c neuron showing spontaneous sodium-mediated action potentials (blocked by TTX, 1.5 μM), as well as spontaneous GABA- and glutamate-mediated postsynaptic potentials (blocked by gabazine (20 μM) and NBQX (40 μM)/AP5 (100 μM), respectively. (**k**) By changing the holding potential in the presence of gabazine (20 μM), AP5 (100 μM), NBQX (40 μM) and TTX (1.5 μM), the receptor potential decreased in amplitude to finally become reversed at +30 mV. (**l**) Motoneurons (upper trace) or glia-like CSF-c cells (lower trace) did not show any response to pressure pulse stimulation. BDA, biotinylated dextran amine; cc, central canal. Scale bars, 10 μm.

The CSF-c neurons have bulb-like protrusions into the central canal^3^, and when these neurons were retrogradely filled from the lateral margin of the spinal cord, a cilium-like structure was observed extending from the bulb-like ending (Fig. 1a, arrowhead). To verify that this structure corresponded to a cilium we showed that it is immunoreactive towards α-tubulin, a protein characteristic of cilia (Fig. 1b arrowhead; see also Supplementary Fig. 1). When the central canal was exposed, as required for patching CSF-c neurons (see Fig. 1c), movements of cilia associated with the bulb-like endings could be detected at high magnification (Supplementary Movie 1).

To explore whether CSF-c neurons are capable of sensing fluid movements, brief pressure pulses were applied to a Ringer-filled micropipette positioned close to the CSF-c cell´s bulb-like protrusion into the central canal, while performing patch recordings of the same cell (Fig. 1c). Fluid pulses (duration 10–80 ms) reliably elicited graded receptor- or action potential responses in all CSF-c neurons tested (*n*=30; Fig. 1d,e), with a short and constant latency. The average response latency was ∼20 ms, irrespective of the duration of the fluid pulse (Fig. 1f). The receptor potential amplitude increased with increasing pulse magnitudes (from 5 to 15 p.s.i. in Fig. 1g), and action potentials were evoked with somewhat higher pulse pressure (20 p.s.i.; Fig. 1g). Figure 1h illustrates the mean increase in receptor potential amplitude for all cells tested (*n*=6), in response to increasing fluid pulse magnitudes.

To verify that the fluid pulse-evoked responses were not indirect or synaptically evoked, we blocked the GABAergic and glutamatergic synaptic transmission by bath-application of gabazine, AP5 and NBQX (Fig. 1i). CSF-c neurons generally show a low level of spontaneous, sodium-mediated action potentials as well as spontaneous GABAergic and glutamatergic postsynaptic potentials^3^, the latter mediated by both α-amino-3-hydroxy-5-methyl-4-isoxazolepropionic acid (AMPA) and *N*-Methyl-D-aspartic acid (NMDA) receptors (Fig. 1j). The responses to pressure pulse stimulation remained unchanged after receptor blockade. Furthermore, to exclude the possibility that the responses in CSF-c neurons could be mediated by other synaptic interactions, recordings were also made in the presence of tetrodotoxin (TTX; together with GABA- and glutamate receptor blockade) and, as shown in Fig. 1k, receptor potential responses to fluid pulses were maintained. This was confirmed in all cells tested (*n*=6). Moreover, by changing the holding potential from −45 to −25 mV the receptor potential decreased in amplitude to finally become reversed at +30 mV (Fig. 1k; see below regarding ASIC3). These findings thus show that direct mechanical activation of the CSF-c neurons is responsible for the evoked responses. In contrast, glia-like CSF-c cells, that also extend a central process into the central canal and a lateral process reaching the stretch receptor neurons, did not show any response to pressure pulse stimulation (*n*=11; Fig. 1l, lower trace). Similarly, motor neurons with dendrites near the midline^10^ did not show a response to fluid pulse stimulation (Fig. 1l, upper trace).

**Figure 2:**
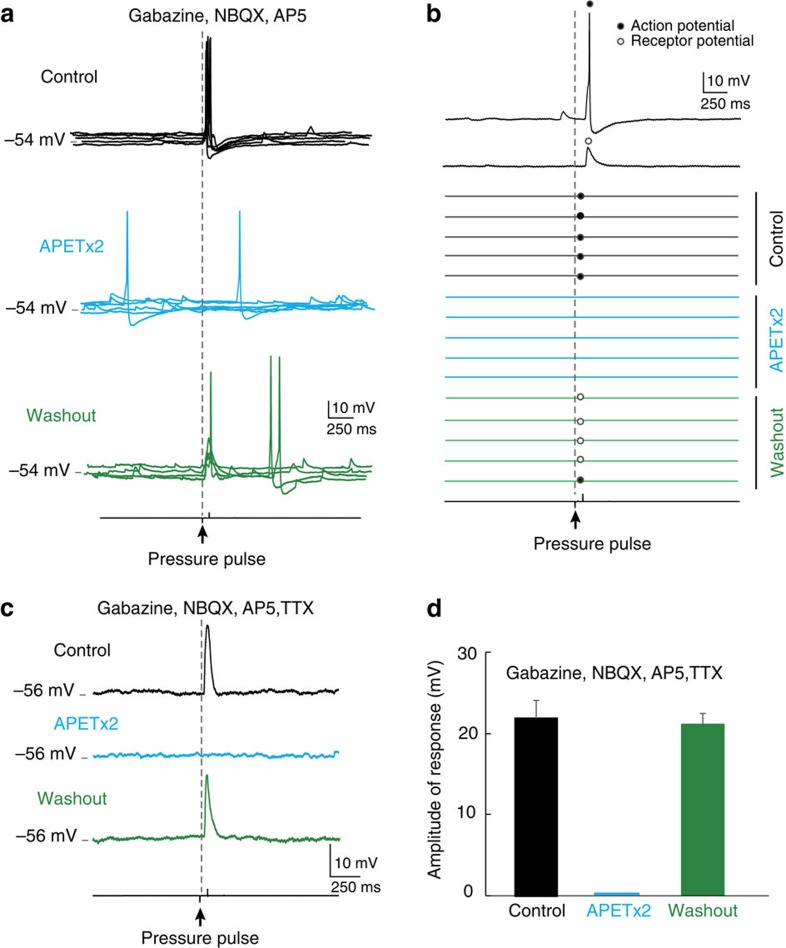
APETx2 inhibits the mechanosensitivity of CSF-c neurons. (**a**) Fluid-pulse stimulation (20 p.s.i., 80 ms) elicited action potential responses in control conditions (black) in the presence of gabazine (20 μM), AP5 (100 μM) and NBQX (40 μM). Application of the ASIC3 blocker APETx2 (2 μM) abolished responses (blue), which partially recovered upon washout (green). (**b**) Raster plot showing reliable responses to pressure stimulations (20 p.s.i., 80 ms) in control, and which were completely abolished by application of APETx2. Responses reappeared upon washout. (**c**) The receptor potential elicited by fluid pulses (20 p.s.i., 60 ms) was also blocked by APETx2 in the presence of TTX (1.5 μM). (**d**) Complete blockade of responses after application of APETx2 (*n*=6). Means±s.e.m.

*Specific blockade of ASIC3 inhibits the mechanosensitivity of CSF-c neurons*. The next question was to identify the ion channel responsible for the mechanotransduction underlying the fluid pulse response. The ASICs are members of the degenerin/epithelial sodium channel (DEG/ENaC) family^11^ and have in addition to being pH sensitive also been shown to be mechanosensitive^12^,^13^,^14^,^15^,^16^ preferentially permeable to Na^+^ ions^17^,^18^. Among the three ASIC subtypes identified^17^, ASIC3 is expressed in sensory neurons and nerve endings^19^,^20^,^21^ and is associated with multimodal sensory perception^14^. To investigate whether ASIC3 could be involved in the mechanosensitivity of CSF-c neurons in lamprey, we bath-applied APETx2, an ASIC3-specific blocker^22^,^23^, while recording the mechanical response to fluid pulses. In all cells tested (*n*=15), the response was eliminated in the presence of APETx2 (Fig. 2). Upon washout, a partial recovery occurred with subthreshold responses, and after a longer washout, complete recovery with spike responses was observed (Fig. 2a,b). The raster plot in Fig. 2b shows that all stimulation pulses reliably elicited action potentials, which were completely abolished by application of APETx2. Also in the presence of TTX, the receptor potential elicited by the fluid pulse was completely blocked by APETx2 (Fig. 2c). Figure 2d illustrates the complete blockade of receptor potential responses in all cells tested (*n*=6).

Thus, the mechanosensitivity of lamprey spinal CSF-c neurons, underlying their response to fluid movements, appears to be mediated by ASIC3.

### CSF-c neurons are sensitive to decreases in extracellular pH

**Figure 3:**
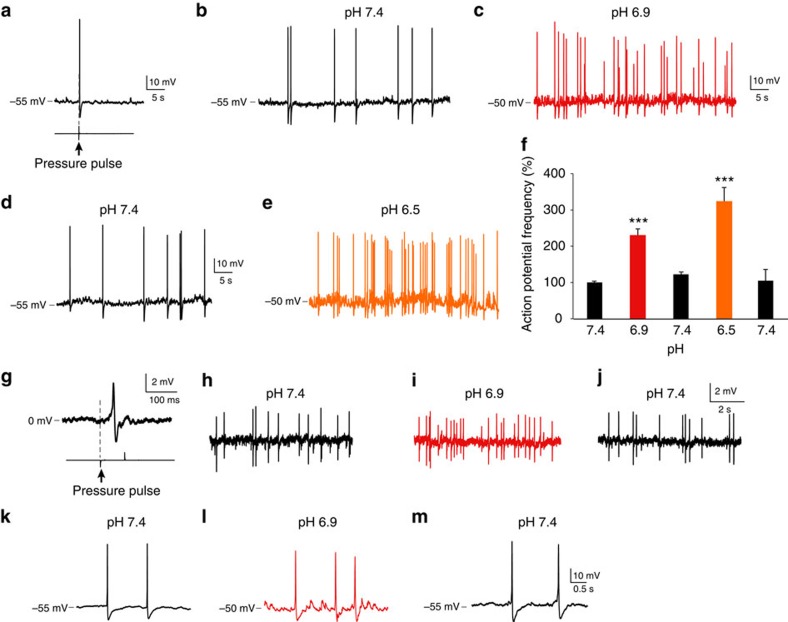
CSF-c neurons are sensitive to pH changes. (**a**) Whole-cell patch recording of a CSF-c neuron, showing mechanosensitivity to a fluid-pulse (20 p.s.i., 80 ms). (**b**) The same neuron firing spontaneous action potentials in control conditions (pH 7.4) in the presence of gabazine (20 μM), AP5 (100 μM) and NBQX (40 μM). (**c**–**e**) Lowering pH to 6.9 increased action potential frequency (**c**; red), and more so at pH 6.5 (**e**; orange) Also, the membrane potential was depolarized by ∼5 mV at lowered pH. (**f**) Action potential frequency during 1 min in CSF-c neurons at pH 7.4, 6.9 and 6.5, respectively. The values are means±s.e.m., normalized to basal activity at pH 7.4 (*n*=15). Student's *t*-test: ***P<0.001, significant difference compared with control, pH 7.4. (**g**) Cell-attached patch recording of a CSF-c neuron showing the response to fluid-pulse stimulation. (**h**–**j**) This mechanosensitive CSF-c neuron in addition responded by increased action potential frequency to lowered pH (6.9), also evident in the cell-attached recording configuration. (**k**–**m**) In addition, subthreshold depolarizing receptor potentials appeared with lowered pH (pH 6.9).

**Figure 4:**
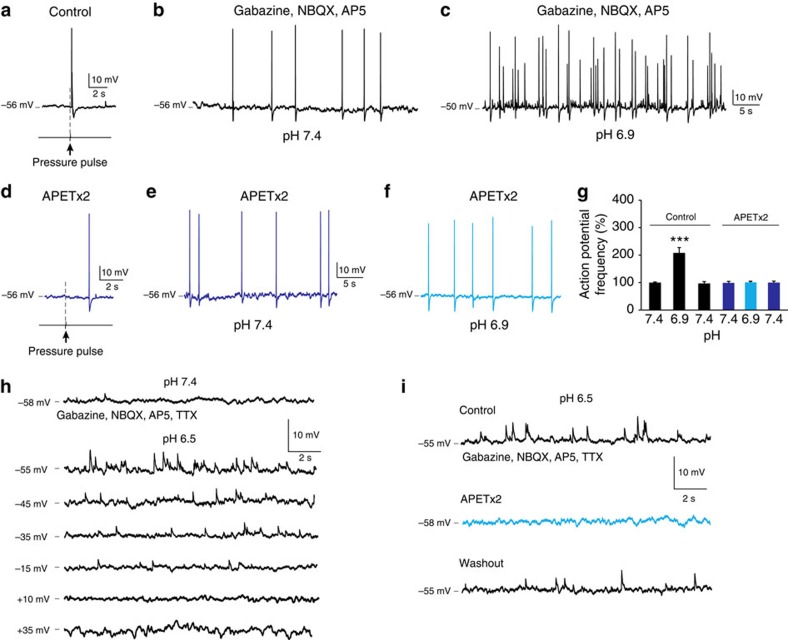
APETx2 inhibits chemosensitivity of CSF-c neurons. (**a**) CSF-c neuron showing mechanosensitivity to a fluid pressure pulse (80 ms, 20 p.s.i.). (**b**,**c**) In the same neuron, the action potential frequency increased at pH 6.9 in the presence of gabazine (20 μM), AP5 (100 μM) and NBQX (40 μM). (**d**) Application of APETx2 (2 μM) abolished the response to fluid-pulse stimulation. (**e**,**f**) APETx2 also abolished the response to pH lowering. (**g**) Firing of CSF-c neurons before and after application of APETx2. The values are means±s.e.m. during 1 min of recording, normalized to basal activity at pH 7.4 (*n*=5). Student's *t*-test: ***P<0.001, significant difference at pH 6.9 compared with 7.4 only in control conditions in the absence of APETx2. (**h**) Decreases in pH resulted in depolarizing potentials also in the presence of TTX (1.5 μM), that reversed at positive holding potentials. (**i**) Addition of APETx2 (1 μM) completely blocked the response to lowered pH in the presence of TTX (1.5 μM).

**Figure 5:**
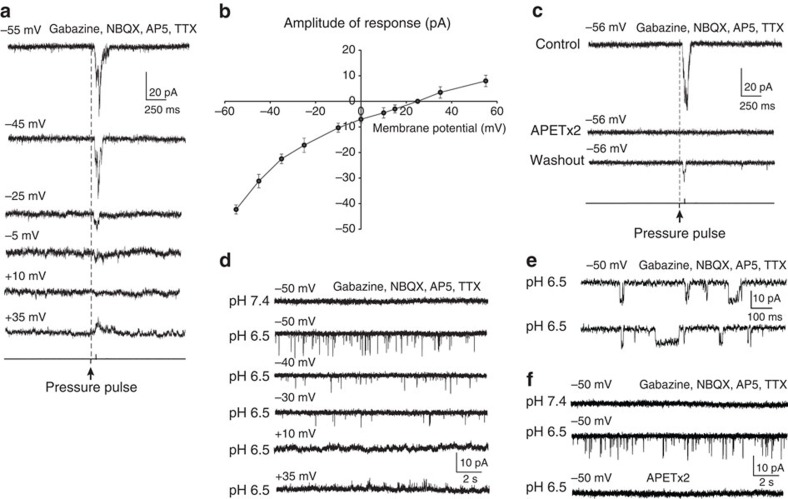
Voltage clamp analysis of the response to fluid pulse stimulation and to decreases in extracellular pH. (**a**) The amplitude of the receptor current increases at hyperpolarised holding potentials and decreases upon depolarization in the presence of gabazine (20 μM), AP5 (100 μM), NBQX (40 μM) and TTX (1.5 μM). (**b**) The receptor current reversed at ∼+25 mV (*n*=5). (**c**) The receptor current evoked by fluid pulse was eliminated in the presence of the ASIC3 blocker APETx2 (1 μM) and returned after washout. The receptor currents in **a**–**c** were elicited by a fluid pulse of 60 ms, 20 p.s.i. (*n*=6). (**d**) No current events were seen at pH 7.4 in the presence of gabazine (20 μM), AP5 (100 μM), NBQX (40 μM) and TTX (1.5 μM). After a decrease in extracellular pH to 6.5, inward current deflections appeared that decreased in amplitude and frequency at more depolarized holding potentials and were reversed in sign at +35 mV (*n*=5). (**e**) At pH 6.5 discrete current deflections were recorded, which may correspond to single-channel openings. (**f**) The inward currents recorded at pH 6.5 were completely blocked in the presence of APETx2 (1 μM; *n*=3). The data in **b** are represented as means±s.e.m.

ASIC3 is also the most chemosensitive of the subtypes of ASICs and is activated by protons upon a modest decrease (7.4>pH>6.5) of the extracellular pH (refs 21, 24), and in contrast to ASIC1 and ASIC2 they show a sustained activation^14^,^25^,^26^. To explore whether CSF-c neurons also respond to changes in pH, we bath-applied extracellular solutions of low pH (from pH 7.4 to 6.9 or 6.5), while performing patch recordings (Fig. 3). To avoid the possibility of indirect effects the synaptic inputs were in all cases blocked by addition of gabazine, AP5 and NBQX. We initially applied fluid pulse stimulation of the cell to confirm the mechanosensitivity of the recorded cells (Fig. 3a,g). At rest, CSF-c neurons fire spontaneous action potentials (Fig. 3b)^3^. When the neurons were exposed to pH 6.9 the frequency of the spontaneous action potentials reversibly increased (Fig. 3c,d), to increase even further at pH 6.5 (Fig. 3e). The increased activity at lowered pH was sustained. This sensitivity to decreases in pH was seen in all CSF-c neurons tested (*n*=15; Fig. 3f). To rule out any interference with the intracellular fluid in the response, we also recorded in cell-attached mode. Figure 3h–j shows that lowered extracellular pH increased the spike frequency also in this configuration. In addition to the increase in spike frequency, a net depolarization of the resting membrane potential of ∼5 mV, as well as a number of subthreshold brief depolarizing potentials were observed with lowered pH (Fig. 3c,e,k–m; see also Figs 4h and 5d,e). These results demonstrate that laterally projecting CSF-c neurons in the lamprey spinal cord, in addition to sensing fluid movements, also respond to decreases in extracellular pH. In contrast, glia-like CSF-c cells (*n*=6) did not show any responses when exposed to pH 6.5 (not illustrated).

*Specific blockade of ASIC3 inhibits the pH-sensitivity of CSF-c neurons*. To investigate if the sensitivity of CSF-c neurons to pH alterations also depends on the ASIC3 subtype, APETx2 was applied. The cell in Fig. 4a–f showed clear responses to both brief pulses of fluid jets (Fig. 4a), and also to lowering of the extracellular pH (Fig. 4b,c). Following a blockade of ASIC3 with APETx2, both the mechanical response and the responses to lowered pH were eliminated (Fig. 4d–f). The dependence on ASIC3 for the pH sensitivity was seen in all CSF-c neurons tested (*n*=5; Fig. 4g). Thus, lowering the pH will lead to activation of ASIC3 in CSF-c neurons. In the presence of TTX, a lowering of pH resulted in the occurrence of depolarizing potentials (*n*=5; Fig. 4h; cf. Figs 3l and 5d,e), as well as a net depolarization of the membrane potential (3 mV in the cell in Fig. 4h). Thus, the chemosensitivity to changes in pH is also because of a direct effect on the CSF-c neurons, and is not indirectly mediated via cellular or synaptic interactions. Furthermore, addition of APETx2 completely blocked the depolarizing potentials as well as the net depolarization seen with lowered pH also in the presence of TTX (*n*=3; Fig. 4i). As with fluid pulse stimulation (Fig. 1k), the depolarizing potentials occurring at lowered pH were reduced in amplitude upon membrane depolarization, to become reversed at positive holding potentials (Fig. 4h).

### Voltage clamp analysis of receptor currents in CSF-c neurons

To characterize the nature of the receptor currents underlying the fluid pulse response, patch recordings from CSF-c neurons were also performed in voltage clamp mode (Fig. 5a–c). The amplitude of the receptor current increased at more hyperpolarized holding potentials, and decreased upon depolarization (Fig. 5a). The receptor current reversed at ∼+25 mV holding potential (Fig. 5b; *n*=5), indicating an involvement of a current carried preferentially by sodium ions, and as reported for ASIC3 in other species^18^,^25^,^27^. Furthermore, the receptor current evoked by the fluid pulse was eliminated in the presence of the ASIC3 blocker (Fig. 5c; *n*=6).

The response to lowered pH was also analysed in voltage clamp mode (Fig. 5d–f). In the presence of GABA- and glutamate receptor blockers (gabazine, AP5 and NBQX) as well as TTX, no current events were seen at normal pH (7.4; Fig. 5d, top trace). After lowering the extracellular pH to 6.5, inward current deflections appeared (Fig. 5d–f) in all cells tested (*n*=5). These inward current events decreased in frequency and amplitude at more depolarized holding potentials, and had reversed sign at +35 mV (Fig. 5d). The discrete current deflections presumably correspond to single-channel openings (Fig. 5e). The inward currents recorded at low pH were completely blocked in the presence of APETx2 (Fig. 5f; *n*=3), indicating that they were generated by the activation of ASIC3 in response to the decrease in pH.

### Activation of CSF-c neurons influence the locomotor network

**Figure 6:**
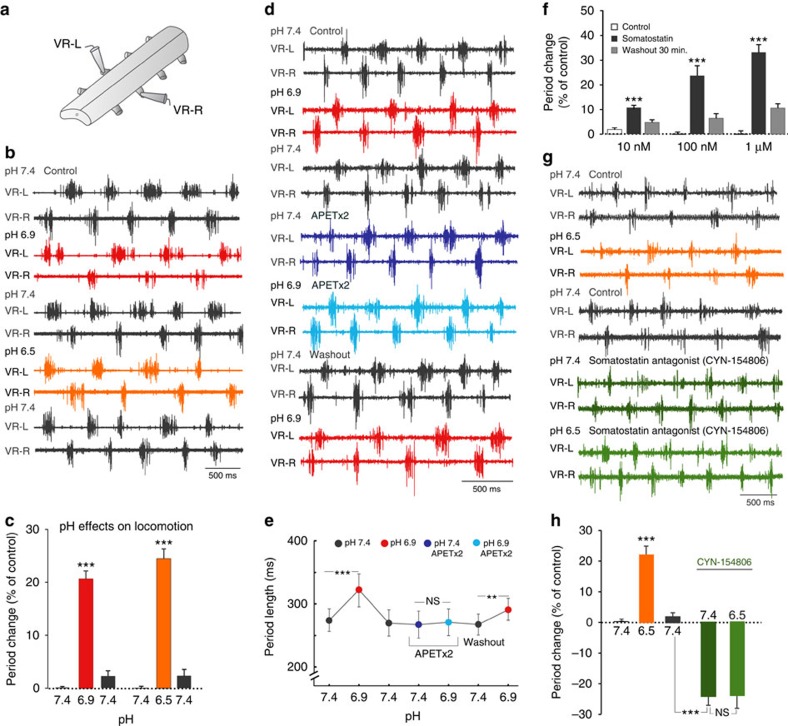
Lowering of extracellular pH or application of somatostatin reduces the locomotor rhythm. (**a**) Illustration of arrangement for ventral root recordings with suction electrodes in the intact, isolated spinal cord preparation (VR-L, VR-R, left and right side ventral root, respectively). (**b**) Bilateral ventral root recording during NMDA (100 μM)-induced fictive locomotion in the isolated lamprey spinal cord, during control conditions (pH 7.4; black traces) and during lowered pH of 6.9 (red traces) and 6.5 (orange traces). (**c**) Decreases in extracellular pH prolonged the cycle period. The mean period was determined during 20 cycles for each of the conditions and normalized to the value during control conditions (% of control; *n*=7). (**d**) Bilateral ventral root recording during control conditions (pH 7.4; black traces), during pH 6.9 (red traces) and in the presence of the ASIC3 blocker APETx2 (1 μM; blue traces). (**e**) Application of APETx2 blocked the effect of lowered extracellular pH (6.9) on the cycle period, which recovered upon washout (mean values calculated for 20 cycles during each condition; *n*=2 preparations). (**f**) Effect of somatostatin (10 nM, 100 nM and 1μΜ) on the cycle period of the locomotor activity. Somatostatin significantly increased the period at all tested concentrations (*n*=19). (**g**) Bilateral ventral root recording in the isolated spinal cord, during control conditions (pH 7.4; black traces), during pH 6.5 (orange traces) and following application of the somatostatin receptor sst_2_ antagonist CYN-154806 (2 μM; green traces). (**h**) Application of CYN-154806 lead to a shortening of the period length at control pH 7.4 (dark green; *n*=4). In the presence of the antagonist, a decrease of pH (here to 6.5; light green) had no effect on the cycle period. The data are represented as means±s.e.m. (**c**,**f,h**) and ±s.d. (**e**); Student's *t*-test: ***P<0.001; **P<0.01, significant difference compared with control; NS: non-significant, ^NS^*P*>0.5.

The laterally projecting CSF-c neurons may indirectly influence the locomotor network through modulation of the stretch receptor neurons^6^, which not only provide movement-related feedback to the central pattern generator^28^ (see also ref. 29), but they may also exert direct effects on neurons of the network since their axons ramify in the grey matter^3^. To investigate this latter possibility, we examined the effect of pH changes in the intact, isolated lamprey spinal cord preparation during fictive locomotor activity (recorded from ventral roots; Fig. 6a,b) induced by bath application of NMDA^30^. A decrease of the extracellular pH from 7.4 to 6.9 or 6.5 caused a marked prolongation of the cycle period, that is, a reduction of the locomotor burst frequency (Fig. 6b,c). Lowering the pH resulted in a significant increase of cycle period in all of seven tested preparations, to recover upon return to normal pH (Fig. 6c). As described above, a lowered pH excites CSF-c neurons via activation of ASIC3 and we therefore bath-applied APETx2 during fictive locomotion, at normal and lowered pH (Fig. 6d). Before addition of the blocker, the locomotor period length was again increased by acidification (pH 6.9), and this effect was blocked by APETx2 and recovered upon washout (Fig. 6d,e). Blockade of ASIC3 completely abolished the pH-effect, and thus the slowing of the locomotor rhythm at moderately lowered extracellular pH appears to be mediated by CSF-c neurons through activation of ASIC3.

CSF-c neurons express both GABA and somatostatin^3^,^6^, and we have previously shown that GABA, which is represented within several spinal systems, will reduce the level of locomotor activity through both GABA_A_ and GABA_B_ with an action on the soma as well as at the presynaptic level, resulting in a decrease of the burst frequency with maintained burst proportion^31^. To examine the effect of somatostatin on the fictive locomotor rhythm, we bath-applied somatostatin (10 nM–1 μΜ; *n*=19; Fig. 6f), which also markedly slowed down the locomotor burst rate in a dose-dependent manner.

Given that CSF-c neurons constitute the only cell-type that expresses somatostatin in the lamprey spinal cord^3^,^6^, and that CSF-c neurons are activated by lowered pH, we examined whether the influence of a pH-decrease on the fictive locomotor rhythm (Fig. 6b,c) could be mediated via a somatostatin release, by applying the somatostatin sst_2_ receptor antagonist CYN-154806 during fictive locomotion. This led to a shortening of the period length, indicating that there may be a tonic release of somatostatin under these conditions (Fig. 6g; *n*=4). Moreover, with the somatostatin antagonist present a lowering of the pH from 7.4 to 6.5 no longer influenced the burst rate (Fig. 6h, right). These results further support the notion that the pH effects on the locomotor activity may originate from a modulation exerted by the pH-sensitive CSF-c neurons.

Since CSF-c neurons receive spontaneous GABAergic IPSPs (but not glycinergic) and glutamatergic EPSPs^3^ (Fig. 1j), we also examined whether they receive phasic input during fictive locomotion (*n*=6; not illustrated), however, with negative results. This parallels findings in the paralysed larval zebrafish^32^. Furthermore, activation of reticulospinal axons (*n*=6) did neither elicit synaptic effects in CSF-c neurons. This spontaneous synaptic activity may thus originate from one of several GABAergic populations in the spinal cord and from non-locomotor-related glutamatergic neurons.

### Bending movement of the spinal cord activates CSF-c neurons

**Figure 7:**
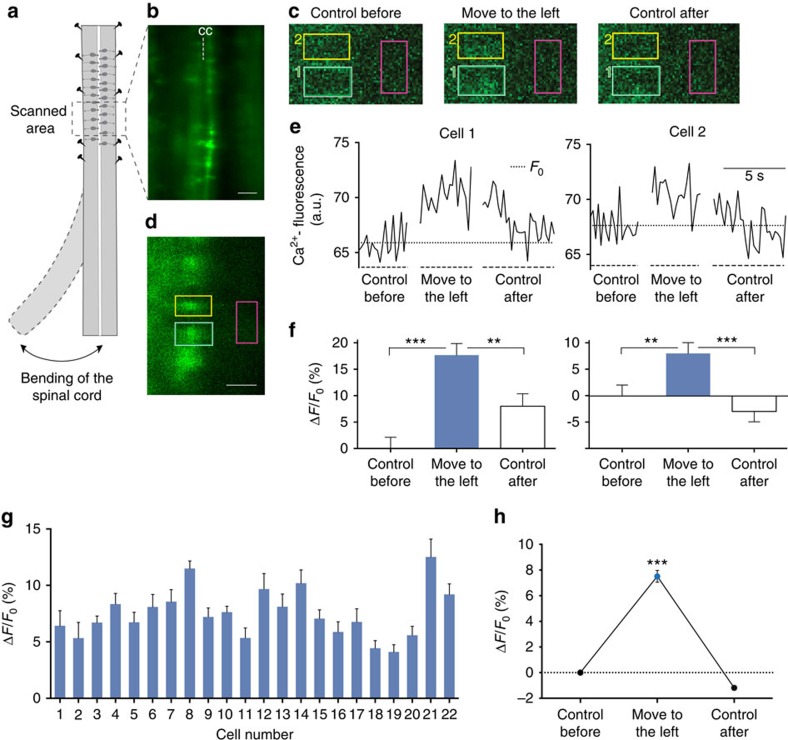
Calcium-imaging during imposed movement of the spinal cord. (**a**) Schematic drawing illustrating the experimental design, with the movable, caudal part of the isolated spinal cord and the rostral part firmly fixed. Live imaging was performed in the area indicated. (**b**) fluorescence overview image within the scanned area, showing two rows of retrogradely filled CSF-c cells on either side of the central canal (cc). (**c**) Image frames from scanning (5 frames per second) before imposing a bending movement (control before), during bending to the left, and after return to the control position (control after). Two measurement regions placed over two CSF-c neurons (green and yellow) are indicated, as well as a reference region for measuring background fluorescence (red). Increased fluorescence intensity can be seen during bending in both cells. (**d**) Overview image from the same preparation as in **c**, with the corresponding measurement regions indicated. Image scanned at slow frame rate with higher spatial resolution and longer pixel dwell time to better visualize the cells recorded from. (**e**) Ca^2+^ fluorescence traces of the cell regions in **c**,**d** during scanning in the control position, during lateral bending and after return to the control position. Dotted line indicates the mean baseline fluorescence level before movement (*F*_0_). (**f**) The increase of fluorescence, (Δ*F/F*_0_), was highly significant in both CSF-c neurons following the bending movement (cell 1: ***P<0.0001; cell 2: **P<0.01; Student's *t*-test). After return to the control position, mean fluorescence intensity significantly decreased in both cells. (**g**) Corresponding data from 22 neurons are analysed, with significantly increased fluorescence intensities (Δ*F/F*_0_) during bending in each cell. Student's *t*-test: *P*<0.05 to *P*<0.0001. (**h**) Pooled data from all cells, showing a significant mean increase in fluorescence (7.5%; Δ*F*/*F*_0_) during bending of the spinal cord (***P<0.0001, Student's *t*-test). The data in **f**–**h** are represented as means±s.e.m. Scale bars, (**b**) 50 μm; (**d**) 20 μm.

Having established that CSF-c neurons are mechanosensitive and respond to applied fluid pulses, we then investigated whether a stimulus involving a lateral bending movement of the intact spinal cord, as during active swimming, would also activate CSF-c neurons (Fig. 7). Cells were retrogradely filled with a fluorescent calcium-indicator (Oregon Green Bapta-1 dextran) from the lateral margin, and a portion of intact spinal cord was then isolated and mounted on a confocal live-imaging system (see Methods). The caudal part of the spinal cord could be moved laterally from a neutral position, while changes of fluorescence in individual CSF-c neurons were recorded by imaging cells in the rostral, fixed part of the spinal cord (Fig. 7a). Figure 7b shows the two rows of retrogradely filled CSF-c cells on either side of the central canal (cc) in the area being scanned. Imaging was performed at 4–6 frames per second, first with the caudal part in the initial, straight position, then with the caudal part in the bent position directly following a movement to the left, and finally after return to the straight position. In the event that any minor displacement of the tissue being scanned was detected, the affected image sequences were omitted from the analysis (see Methods). Figure 7c shows examples of image frames recorded in the straight position (control before), following the lateral bending movement (move to the left), and after return to the straight position (control after), and with two measurement regions over two neurons (green and yellow boxes), together with a region for measuring background fluorescence (red box). The corresponding regions are also indicated in an overview image at higher spatial resolution in Fig. 7d. Increased fluorescence intensity could be detected for both cells during bending of the spinal cord (Fig. 7e), with the traces during bending reaching values well above the baseline level recorded in the control position (*F*_0_, dotted lines). A gradual return of fluorescence intensities was seen upon return to the straight position. The mean increase of Ca^2+^-fluorescence during lateral bending (Δ*F/F*_0_), was highly significant in both CSF-c neurons (Fig. 7f). Upon return to the control position, the mean fluorescence intensity significantly decreased in both cells (in cell 2 even below the initial baseline value). Corresponding results were found in all 22 neurons analysed (Fig. 7g), with a significant increase of fluorescence intensity (Δ*F/F*_0_) in each cell, and the pooled data from all cells revealed a highly significant increase of 7.5% in fluorescence during bending of the spinal cord (*P*<0.0001; Fig. 7h). These findings thus indicate that a lateral bending movement of the spinal cord, as would occur during swimming, may indeed activate CSF-c neurons.

## Discussion

We show that ciliated CSF-c neurons with laterally projecting processes sense both fluid movements and decreases in extracellular pH. The cilia of these neurons express α-tubulin. At the ultrastructural level, GABAergic CSF-c neurons have been shown to have ciliated endings in lamprey and other species^5^,^33^,^34^. The presence of cilia on CSF-c neurons would allow these cells to sense the movements of the CSF within the central canal. When the CSF-c neurons were subjected to brief graded fluid pulses, graded receptor potentials were elicited and finally an action potential. The receptor potential had an equilibrium potential around +25 mV that remained after a blockage of synaptic transmission and in tetrodotoxin, testifying to its origin in the CSF-c neurons themselves. Since the receptor potential was modulated by changes in the holding potential, the remote possibility that these changes were due to an action on nearby cells and mediated by gap junctions can also be excluded.

**Figure 8:**
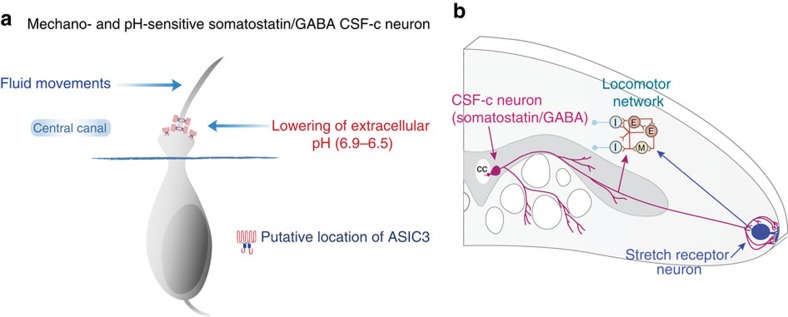
CSF-c neurons sensitive to both fluid movement and lowered pH influence the spinal locomotor network as well as stretch receptor neurons. (**a**) Ciliated CSF-c neurons respond to fluid movements and to lowered pH through activation of ASIC3. (**b**) Schematic illustration of a cross-section of the lamprey spinal cord, with a CSF-c neuron by the central canal (cc) and lateral axonal projections in the grey matter providing input to the locomotor network and projections in close apposition to a stretch receptor neuron at the spinal cord margin.

Which ion channels mediate the receptor potential in CSF-c neurons? Acid-sensing ion channels have in several studies been shown to function as mechanotransducers^13^,^35^,^36^ in addition to being activated by protons upon a moderate decrease of the extracellular pH (refs 14, 20, 21, 37). The pH sensitivity varies, however, across the ASICs. ASIC3 is activated at pH 6.9–6.4 (refs 17, 37), the range at which the CSF-c neurons were activated. By applying APETx2, the ASIC3-specific blocker, we show that both the mechanical response to fluid movements and the response to a pH decrease were eliminated in the CSF-c neurons, suggesting that these effects are both mediated by ASIC3 channels (Fig. 8a). Our finding of a reversal potential at +25 mV also agrees well with previous reports on sodium-selective ASIC3 channels in other species^18^,^25^,^27^. Among the three ASIC subtypes identified^17^, ASIC3 is widely expressed in sensory neurons and nerve endings^19^,^20^,^21^,^36^, sites where mechanical and noxious stimuli are converted into electrical signals. ASIC3 channels are present in both the peripheral and central nervous systems^35^,^38^,^39^.

GABAergic CSF-c neurons in the teleost and mammalian spinal cord also express the transient receptor potential channel PKD2L1 (ref. 40), which can trigger action potentials in response to decreases in pH (ref. 41). However, recent studies demonstrate that PKD2L1 channels are instead activated by increases in pH (alkalization^42^,^43^), and respond to acid stimuli with an off-response^44^,^45^ and thus that an alkali-activated mechanism is responsible for eliciting the off-response^42^. This is in contrast to the present results in which the CSF-c neurons respond to a small decrease of the pH, and which also show that the proton-induced receptor potentials only occur under these conditions and are maintained as long as the pH is low. Indeed, a lowering of pH activates a sustained current in ASIC3 channels^14^. In addition, acidic pH evoked a current in PKD2L1-expressing spinal CSF-c neurons that displayed characteristics of ASICs^46^. A specific contribution of ASIC3 channels to the pH-sensitivity of CSF-c neurons has not been tested in mammals.

The GABAergic CSF-c neurons are the only cells that express somatostatin in the spinal cord^3^,^6^. Their axons ramify in the grey matter and they also inhibit the stretch receptor neurons at the lateral margin, through both GABA and somatostatin^6^. The latter form an integrated part of the locomotor system by sensing the undulatory movements during locomotion and providing feedback to the locomotor network (Fig. 8b).

We show that somatostatin has a depressing effect on the locomotor burst rate, as already known for GABA^31^,^47^. Moreover, applying a somatostatin antagonist during fictive locomotion results in a frequency increase, suggesting that there is indeed a release of somatostatin, presumably from tonically active CSF-c neurons. As important, the decrease of the locomotor burst frequency induced by lowering the pH is blocked by administering a somatostatin antagonist. These data taken together provide evidence that CSF-c neurons indeed have a direct modulatory effect on the locomotor network itself (see Fig. 8b).

What could be the role of the CSF-c neurons in the control of motion? An enhanced level of activity in different parts of the CNS will lead to a release of lactate, which in turn will lower the pH (refs 48, 49). Decreases in brain pH to 6.5 or below occur, for example, in cerebral ischaemia, hypoxia or epilepsy due to increased lactate concentrations during glycolysis^50^,^51^, and also neuronal activity during normal function can lower the pH (ref. 52). This will consequently result in acidosis and disrupt pH homoeostasis if the protons are not cleared. A modest acidification down to pH 6.9–6.5 will result in persistent activation of ASIC3 (ref. 17). In the case of the lamprey spinal cord a high level of neuronal activity would thus be expected to enhance the levels of lactate and thereby lead to a further activation of the CSF-c neurons through ASIC3. This would provide a negative feedback and lower the degree of activity in the locomotor network. We show here that the depressing effect on the locomotor rhythm by lowered pH is indeed mediated by ASIC3. We also show that the CSF-c neurons are activated during movement through their mechanosensitivity, which in a similar way will act to reduce the degree of activation of the network. The two signals could thus add to each other. For instance, if there is a high level of neural activity (lowering the pH) and a high level of locomotor activity activating the movement sensors, these two signals would be synergistic and be complementary. This would also provide a logic for having the ASIC3 receptors be sensitive to both a reduced pH and motion. The role of CSF-c neurons seems therefore to be to dampen the degree of activity—or perhaps rather prevent over-excitation. This is also in line with the previous demonstration in zebrafish showing that an optogenetic activation of central canal neurons can lead to a depression of the locomotor activity^9^. In the case of the zebrafish, however, the mechanism for activation of CSF-c neurons has remained elusive.

## Methods

### Animals

Experiments were performed on a total of 84 adult river lampreys (*Lampetra fluviatilis*) of both sexes that were collected from the Ljusnan River, Hälsingland, Sweden. The experimental procedures were approved by the local ethical committee (Stockholm's Norra Djurförsöksetiska Nämnd) and were in accordance with The Guide for the Care and Use of Laboratory Animals (National Institutes of Health, 1996 revision). During the investigation, every effort was made to minimize animal suffering and to reduce the number of animals used.

### Retrograde tracing and immunohistochemistry

The animals (*n*=6) were deeply anesthetised through immersion in fresh water containing tricane methanesulfonate (MS-222; 100 mg l^−1^; Sigma, St Louis, MO, USA). Following decapitation, a 2 cm long portion of the spinal cord, rostral to the dorsal fin, was dissected out and immersed in ice-cooled oxygenated HEPES-buffered physiological solution containing (in mM): 138 NaCl, 2.1 KCl, 1.8 CaCl_2_, 1.2 MgCl_2_, 4 glucose, 2 HEPES, and with pH adjusted to 7.4 with NaOH. Laterally projecting CSF-c cells were retrogradely labelled by injection of biotinylated dextran amine (BDA, 20% in distal water containing Fast Green to aid visualization; Molecular Probes Europe BV, Leiden, The Netherlands) into the lateral edge of the isolated spinal cord. Injections (∼30 nl) were made with a glass micropipette (borosilicate, OD=1.5 mm, ID=1.17 mm; Hilgenberg GmbH, Malsfeld, Germany), with a tip diameter of 10–20 μm. The micropipette was fixed in a Narishige micromanipulator holder and attached to an air supply for pressure pulse injection. After injection, the spinal cords were kept in the cold room (+4 °C) for 12–24 h. Both intact (*n*=4) and spinal cords subjected to retrograde tracing (*n*=2) were fixed by immersion in 4% formaldehyde and 14% saturated picric acid solution in 0.1 M phosphate buffer (PB) for 12–24 h at 4 °C, and subsequently cryoprotected in 20% sucrose in PB for 3–12 h. Transverse sections (20 μm thick) were cut on a cryostat (Microm HM 560, Microm International GmbH, Walldorf, Germany), collected on gelatin-coated slides and kept at −20 °C until processing.

For immunohistochemical detection of α-tubulin in retrogradely labelled CSF-c neurons, sections were incubated overnight with a mouse monoclonal anti-acetylated α-tubulin antibody (dilution 1:500; Sigma-Aldrich). The sections were subsequently rinsed thoroughly in 0.01 M phosphate-buffered saline and incubated with a mixture of Alexa Fluor 488-conjugated streptavidin (1:1,000; Jackson ImmunoResearch, West Grove, PA, USA) and Cy3-conjugated donkey anti-mouse IgG (1:500; Jackson ImmunoResearch) for 2 h. Sections not subjected to tracer injection were co-incubated with the anti-α-tubulin and a rat monoclonal anti-somatostatin antibody (dilution 1:200; Millipore; Bedford, MA, USA), followed by Cy3-conjugated donkey anti-mouse IgG (1:500; Jackson ImmunoResearch) and Alexa Fluor 488-conjugated donkey anti-rat IgG (1:200; Jackson ImmunoResearch). All sections were mounted with glycerol containing 2.5% diazabicyclooctane (Sigma-Aldrich, St Louis, MO, USA). The primary and secondary antibodies were diluted in 1% bovine serum albumin, 0.3% Triton X-100 in 0.1 M PB.

### Electrophysiology

Animals (*n*=49) were deeply anaesthetised as described above and the spinal cord was isolated from the notochord, and the meninx primitiva was removed. The spinal cord was then placed in ice-cooled HEPES-buffered physiological solution (extracellular solution) containing (in mM): 138 NaCl, 2.1 KCl, 1.8 CaCl_2_, 1.2 MgCl_2_, 4 glucose and 2 HEPES, pH 7.4. pH was adjusted to various pH values (7.4, 6.9, 6.5) with NaOH and osmolarity to 290 mOsm l^−1^ with glucose. The dorsal tissue above the central canal was removed by longitudinal slicing to expose the central canal lumen (Micro-slicer DTK-1,000, Japan). The spinal cord preparation was then mounted in a cooled chamber (4–8 °C), continuously perfused with physiological solution and placed on the stage of an upright fluorescence microscope (Olympus BX51W1, Olympus Sverige AB, Stockholm, Sweden). Movements of the cilia that extend into the central canal could be observed in the microscope. Patch electrode and fluid pressure pipettes were prepared from borosilicate glass microcapillaries (Hilgenberg GmbH) using a two-stage puller (PP-830, Narishige, Japan). Patch electrodes (8–12 MΩ) were filled with an intracellular solution of the following composition (in mM): 130 K-gluconate, 5 KCl, 10 HEPES, 4 Mg-ATP, 0.3 Na-GTP and 10 phosphocreatine sodium salt. The pH of the solution was adjusted to 7.4 with KOH and osmolarity to 270 mOsm l^−1^ with water. Fluid pressure pipettes (2–4 MΩ) were filled with the extracellular solution. Fluid pulse stimuli were given by applying 5–20 p.s.i. pressure pulses of 10–80 ms duration by a PicoSpritzer II unit (Parker Hannifin Corporation, NJ, USA).

Cells lateral to the central canal were recorded in whole-cell, or in some cases cell-attached configuration, and in current or voltage clamp mode using an Axoclamp 2B or Multiclamp 700B amplifier (Molecular Devices Corp., CA, USA). Bridge balance and pipette capacitance compensation were adjusted and signals were digitized and recorded using Clampex software and analysed in Clampfit (pCLAMP 10, Molecular Devices, CA, USA). Cells were visualized (Fig. 1c) with differential interference contrast/infrared optics. Resting membrane potentials were determined in current clamp mode during whole-cell recording. Depolarizing steps (−55 to +55 mV) were injected and the currents were monitored in voltage clamp mode. The following drugs were added to the extracellular solution and applied by bath perfusion: the specific ASCI3 blocker APETx2 (1–2 μM, Merck Chemicals Ltd., Nottingham, UK), the GABA_A_ receptor antagonist gabazine (20 μM, Tocris, Ellisville, MO, USA), the NMDA receptor antagonist AP5 (100 μM, Tocris), the AMPA receptor antagonist NBQX (40 μM, Tocris) and TTX (1.5 μM; Sigma).

### Fictive swimming experiments

Spinal cord-notochord preparations (10–20 segments; *n*=28) were dissected from the area between the gills and the dorsal fin as described previously^53^ and pinned down in a Sylgard-lined chamber continuously perfused with oxygenated physiological solution which was kept at 8–10 °C. NMDA (100–150 μM, Tocris) was added to the physiological solution to induce fictive locomotion^30^. The motor activity was monitored by the use of glass suction electrodes positioned on two opposite ventral roots (see Fig. 6b). Swimming sequences were analysed for at least 20 cycles recorded during each experimental condition. The effects on the locomotor burst frequency induced by decreases in extracellular pH (pH 6.9 and 6.5) were analysed, as well as the effects of somatostatin (10 nM to 1 μΜ, Tocris). Corresponding experiments were also performed in the presence of the ASIC3 antagonist APETx2 (1 μM) or the somatostatin receptor sst_2_ antagonist CYN-154806 (2 μM; Tocris).

### Calcium-imaging

Injections were made into the lateral spinal cord (*n*=2) with the calcium indicator Oregon Green 488 Bapta-1 dextran (10 kD; 5% in PBS pH 7.4; Molecular Probes), as described above for BDA. The animals were returned to their aquarium for 12 h to allow retrograde labelling of CSF-c cells. The spinal cord was subsequently isolated, the rostral part firmly mounted in a Sylgard-lined open perfusion chamber with the ventral side up, and continuously perfused with HEPES-buffered physiological solution. The caudal part was fixed to a movable portion of the Sylgard lining, which could be moved manually from left to right by the use of forceps (see Fig. 7a). The occurrence of any minor displacement of the tissue being scanned in the rostral part was continuously monitored and directly detected in the recorded image frames. To exclude that any movement artefact could influence the results, image sequences with a tissue displacement were omitted from the analysis. Live imaging of areas comprising labelled CSF-c cells was performed on a Zeiss LSM 510 NLO confocal microscope system (Carl Zeiss, Germany) at 4–6 frames per seconds. Fluorescence emitted by CSF-c cells, designated as regions of interest, was recorded before, during and after bending of the caudal part of the spinal cord (see Fig. 7a). Time-series of fluorescence images from Ca^2+^-imaging experiments were analysed off-line. The average fluorescence intensity of selected regions of interests was analysed using Zeiss LSM software and expressed as relative values in arbitrary units. Changes in Ca^2+^ fluorescence in response to bending movement of the caudal spinal cord (Δ*F/F*_0_) were normalized to the baseline fluorescence recorded with the spinal cord in the initial, straight position (*F*_0_).

### Image analysis

Photomicrographs were taken with an Olympus XM10 digital camera mounted on an Olympus BX51 microscope. Illustrations were prepared in Corel Draw X6 and Adobe Illustrator and Photoshop CS6. Confocal images of tissue sections were obtained using a Zeiss laser-scanning microscope (LSM 510 NLO) and processed using Zeiss LSM software. Images were adjusted only for brightness and contrast.

### Statistical analysis

Data were analysed and presented as means±s.d. or s.e.m., and statistical comparisons were made by using Student's two-tailed *t*-test.

## Additional information

**How to cite this article:** Jalalvand, E. *et al*. Ciliated neurons lining the central canal sense both fluid movement and pH through ASIC3. *Nat. Commun.* 7:10002 doi: 10.1038/ncomms10002 (2016).

## Supplementary Material

Supplementary Information Supplementary Figure 1

Supplementary Movie 1 Cilia movements. Movie sequence captured through the microscope using DIC/IR, showing spontaneous movements of cilia of CSF-c neurons in the central canal (normal frame rate). Two locations with beating cilia are indicated (red lines). Patch electrode placed on one CSF-c neuron is seen below, as well as the pressure pipette used for fluid pulse stimulation (no stimulation given during the sequence).

## References

[b1] Agduhr E. Über ein centrales Sinnesorgan (?) bei den Vertebraten. Z. Anat. Entwickl. Gesch. 66, 223–360 (1922) .

[b2] Dale N., Roberts A., Ottersen O. P. & Storm-Mathisen J. The morphology and distribution of 'Kolmer-Agduhr cells', a class of cerebrospinal-fluid-contacting neurons revealed in the frog embryo spinal cord by GABA immunocytochemistry. Proc. R. Soc. Lond. B Biol. Sci. 232, 193–203 (1987) . 2892204 10.1098/rspb.1987.0068

[b3] Jalalvand E., Robertson B., Wallén P., Hill R. H. & Grillner S. Laterally projecting cerebrospinal fluid-contacting cells in the lamprey spinal cord are of two distinct types. J. Comp. Neurol. 522, 1753–1768 (2014) . 24436002 10.1002/cne.23542

[b4] Kolmer W. Das Sinnesorgan der Wirbeltiere. Z. Anat. Entwickl. Gesch. 60, 652–717 (1921) .

[b5] Vigh B. . The system of cerebrospinal fluid-contacting neurons. Its supposed role in the nonsynaptic signal transmission of the brain. Histol. Histopathol. 19, 607–628 (2004) . 15024719 10.14670/HH-19.607

[b6] Christenson J., Alford S., Grillner S. & Hökfelt T. Co-localized GABA and somatostatin use different ionic mechanisms to hyperpolarize target neurons in the lamprey spinal cord. Neurosci. Lett. 134, 93–97 (1991) . 1687706 10.1016/0304-3940(91)90516-v

[b7] Grillner S., Williams T. & Lagerbäck P. A. The edge cell, a possible intraspinal mechanoreceptor. Science 223, 500–503 (1984) . 6691161 10.1126/science.6691161

[b8] Wyart C. . Optogenetic dissection of a behavioural module in the vertebrate spinal cord. Nature 461, 407–410 (2009) . 19759620 10.1038/nature08323 PMC2770190

[b9] Fidelin K. . CSF-contacting neurons modulate slow locomotion and intersegmental coordination. Society for Neuroscience 828.16 (Neuroscience Meeting Planner, Washington DC, 2014) .

[b10] Wallén P., Grillner S., Feldman J. L. & Bergelt S. Dorsal and ventral myotome motoneurons and their input during fictive locomotion in lamprey. J. Neurosci. 5, 654–661 (1985) . 3973690 10.1523/JNEUROSCI.05-03-00654.1985 PMC6565027

[b11] Waldmann R. & Lazdunski M. H(+)-gated cation channels: neuronal acid sensors in the NaC/DEG family of ion channels. Curr. Opin. Neurobiol. 8, 418–424 (1998) . 9687356 10.1016/s0959-4388(98)80070-6

[b12] Chalfie M. Neurosensory mechanotransduction. Nat. Rev. Mol. Cell. Biol. 10, 44–52 (2009) . 19197331 10.1038/nrm2595

[b13] Delmas P., Hao J. & Rodat-Despoix L. Molecular mechanisms of mechanotransduction in mammalian sensory neurons. Nat. Rev. Neurosci. 12, 139–153 (2011) . 21304548 10.1038/nrn2993

[b14] Li W. G. & Xu T. L. ASIC3 channels in multimodal sensory perception. ACS Chem. Neurosci. 2, 26–37 (2011) . 22778854 10.1021/cn100094b PMC3369706

[b15] Sukharev S. & Corey D. P. Mechanosensitive channels: multiplicity of families and gating paradigms. Sci. STKE re4, 1–24 (2004) . 10.1126/stke.2192004re4 14872099

[b16] Welsh M. J., Price M. P. & Xie J. Biochemical basis of touch perception: mechanosensory function of degenerin/epithelial Na^+^ channels. J. Biol. Chem. 277, 2369–2372 (2002) . 11706013 10.1074/jbc.R100060200

[b17] Deval E. . Acid-sensing ion channels (ASICs): pharmacology and implication in pain. Pharmacol. Ther. 128, 549–558 (2010) . 20807551 10.1016/j.pharmthera.2010.08.006

[b18] Zhang M. . Functional characterization of acid-sensing ion channels in cultured neurons of rat inferior colliculus. Neuroscience 154, 461–472 (2008) . 18456416 10.1016/j.neuroscience.2008.03.040

[b19] Delaunay A. . Human ASIC3 channel dynamically adapts its activity to sense the extracellular pH in both acidic and alkaline directions. Proc. Natl Acad. Sci. USA 109, 13124–13129 (2012) . 22829666 10.1073/pnas.1120350109 PMC3420194

[b20] Lingueglia E. Acid-sensing ion channels in sensory perception. J. Biol. Chem. 282, 17325–17329 (2007) . 17430882 10.1074/jbc.R700011200

[b21] Molliver D. C. . ASIC3, an acid-sensing ion channel, is expressed in metaboreceptive sensory neurons. Mol. Pain 1, 35 (2005) . 16305749 10.1186/1744-8069-1-35 PMC1308857

[b22] Blanchard M. G., Rash L. D. & Kellenberger S. Inhibition of voltage-gated Na(+) currents in sensory neurones by the sea anemone toxin APETx2. Br. J. Pharmacol. 165, 2167–2177 (2012) . 21943094 10.1111/j.1476-5381.2011.01674.x PMC3413854

[b23] Diochot S. . A new sea anemone peptide, APETx2, inhibits ASIC3, a major acid-sensitive channel in sensory neurons. EMBO J. 23, 1516–1525 (2004) . 15044953 10.1038/sj.emboj.7600177 PMC391081

[b24] Holzer P. in Handbook of Experimental Pharmacology eds Canning B. J., Spina D. 283–332Springer-Verlag (2009) . 19655107 10.1007/978-3-540-79090-7_5 PMC7120605

[b25] Waldmann R. . Molecular cloning of a non-inactivating proton-gated Na^+^ channel specific for sensory neurons. J. Biol. Chem. 272, 20975–20978 (1997) . 9261094 10.1074/jbc.272.34.20975

[b26] Yagi J., Wenk H. N., Naves L. A. & McCleskey E. W. Sustained currents through ASIC3 ion channels at the modest pH changes that occur during myocardial ischemia. Circ. Res. 99, 501–509 (2006) . 16873722 10.1161/01.RES.0000238388.79295.4c

[b27] Babinski K., Catarsi S., Biagini G. & Seguela P. Mammalian ASIC2a and ASIC3 subunits co-assemble into heteromeric proton-gated channels sensitive to Gd3^+^. J. Biol. Chem. 275, 28519–28525 (2000) . 10842183 10.1074/jbc.M004114200

[b28] Di Prisco G. V., Wallen P. & Grillner S. Synaptic effects of intraspinal stretch receptor neurons mediating movement-related feedback during locomotion. Brain Res. 530, 161–166 (1990) . 1980227 10.1016/0006-8993(90)90675-2

[b29] Vinay L., Barthe J. Y. & Grillner S. Central modulation of stretch receptor neurons during fictive locomotion in lamprey. J. Neurophysiol. 76, 1224–1235 (1996) . 8871232 10.1152/jn.1996.76.2.1224

[b30] Grillner S., McClellan A., Sigvardt K., Wallén P. & Wilén M. Activation of NMDA-receptors elicits "fictive locomotion" in lamprey spinal cord *in vitro*. Acta. Physiol. Scand. 113, 549–551 (1981) . 6291323 10.1111/j.1748-1716.1981.tb06937.x

[b31] Tegnér J., Matsushima T., el Manira A. & Grillner S. The spinal GABA system modulates burst frequency and intersegmental coordination in the lamprey: differential effects of GABAA and GABAB receptors. J. Neurophysiol. 69, 647–657 (1993) . 8385187 10.1152/jn.1993.69.3.647

[b32] Boehm U. L. . Physiological recruitment of CSF-contacting neurons *in vivo*. Society for Neuroscience 65.20 (Neuroscience Meeting Planner, Washington DC, 2014) .

[b33] Schotland J. L., Shupliakov O., Grillner S. & Brodin L. Synaptic and nonsynaptic monoaminergic neuron systems in the lamprey spinal cord. J. Comp. Neurol. 372, 229–244 (1996) . 8863128 10.1002/(SICI)1096-9861(19960819)372:2<229::AID-CNE6>3.0.CO;2-5

[b34] Vigh-Teichmann I. & Vigh B. The system of cerebrospinal fluid-contacting neurons. Arch. Histol. Jpn 46, 427–468 (1983) . 6362609 10.1679/aohc.46.427

[b35] Fromy B., Lingueglia E., Sigaudo-Roussel D., Saumet J. L. & Lazdunski M. Asic3 is a neuronal mechanosensor for pressure-induced vasodilation that protects against pressure ulcers. Nat. Med. 18, 1205–1207 (2012) . 22842475 10.1038/nm.2844

[b36] Price M. P. . The DRASIC cation channel contributes to the detection of cutaneous touch and acid stimuli in mice. Neuron 32, 1071–1083 (2001) . 11754838 10.1016/s0896-6273(01)00547-5

[b37] Wemmie J. A., Taugher R. J. & Kreple C. J. Acid-sensing ion channels in pain and disease. Nat. Rev. Neurosci. 14, 461–471 (2013) . 23783197 10.1038/nrn3529 PMC4307015

[b38] Del Valle M. E., Cobo T., Cobo J. L. & Vega J. A. Mechanosensory neurons, cutaneous mechanoreceptors, and putative mechanoproteins. Microsc. Res. Tech. 75, 1033–1043 (2012) . 22461425 10.1002/jemt.22028

[b39] Meng Q. Y., Wang W., Chen X. N., Xu T. L. & Zhou J. N. Distribution of acid-sensing ion channel 3 in the rat hypothalamus. Neuroscience 159, 1126–1134 (2009) . 19356693 10.1016/j.neuroscience.2009.01.069

[b40] Djenoune L. . Investigation of spinal cerebrospinal fluid-contacting neurons expressing PKD2L1: evidence for a conserved system from fish to primates. Front. Neuroanat. 8, 26 (2014) . 24834029 10.3389/fnana.2014.00026 PMC4018565

[b41] Huang A. L. . The cells and logic for mammalian sour taste detection. Nature 442, 934–938 (2006) . 16929298 10.1038/nature05084 PMC1571047

[b42] Chen P. . PKD2L1/PKD1L3 channel complex with an alkali-activated mechanism and calcium-dependent inactivation. Eur. Biophys. J. 44, 483–492 (2015) . 26066678 10.1007/s00249-015-1040-y

[b43] Shimizu T., Higuchi T., Fujii T., Nilius B. & Sakai H. Bimodal effect of alkalization on the polycystin transient receptor potential channel, PKD2L1. Pflugers. Arch. 461, 507–513 (2011) . 21340459 10.1007/s00424-011-0934-5

[b44] Inada H. . Off-response property of an acid-activated cation channel complex PKD1L3-PKD2L1. EMBO Rep. 9, 690–697 (2008) . 18535624 10.1038/embor.2008.89 PMC2475332

[b45] Orts-Del'immagine A. . Properties of subependymal cerebrospinal fluid contacting neurones in the dorsal vagal complex of the mouse brainstem. J. Physiol. 590, 3719–3741 (2012) . 22570378 10.1113/jphysiol.2012.227959 PMC3476630

[b46] Bushman J. D., Ye W. & Liman E. R. A proton current associated with sour taste: distribution and functional properties. FASEB J. 29, 3014–3026 (2015) . 25857556 10.1096/fj.14-265694 PMC4763920

[b47] Schmitt D. E., Hill R. H. & Grillner S. The spinal GABAergic system is a strong modulator of burst frequency in the lamprey locomotor network. J. Neurophysiol. 92, 2357–2367 (2004) . 15190090 10.1152/jn.00233.2004

[b48] Magistretti P. J. & Allaman I. A cellular perspective on brain energy metabolism and functional imaging. Neuron 86, 883–901 (2015) . 25996133 10.1016/j.neuron.2015.03.035

[b49] Zeng W. Z. & Xu T. L. Proton production, regulation and pathophysiological roles in the mammalian brain. Neurosci. Bull 28, 1–13 (2012) . 22233885 10.1007/s12264-012-1068-2 PMC5560292

[b50] Rehncrona S. Brain acidosis. Ann. Emerg. Med. 14, 770–776 (1985) . 3927794 10.1016/s0196-0644(85)80055-x

[b51] Siesjo B. K., von Hanwehr R., Nergelius G., Nevander G. & Ingvar M. Extra- and intracellular pH in the brain during seizures and in the recovery period following the arrest of seizure activity. J. Cereb. Blood. Flow. Metab. 5, 47–57 (1985) . 3972923 10.1038/jcbfm.1985.7

[b52] Kaila K. & Chesler M. in pH and Brain Function eds Kaila K., Ransom B. R. 309–337Wiley-Liss (1998) .

[b53] Wallén P. & Williams T. L. Fictive locomotion in the lamprey spinal cord in vitro compared with swimming in the intact and spinal animal. J. Physiol. 347, 225–239 (1984) . 6142945 10.1113/jphysiol.1984.sp015063 PMC1199444

