# Supplementary material for: Ciliated neurons lining the central canal sense both fluid movement and pH through ASIC3
Source: Nat Commun. 2016 Jan 8;7:10002. doi: 10.1038/ncomms10002 (PMC4729841; doi:10.1038/ncomms10002)
Supplement: Supplementary Information — Supplementary Figure 1 [file ncomms10002-s1.pdf]

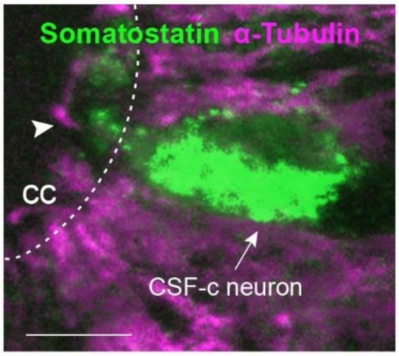

Supplementary Figure 1. Somatostatin immunoreactive CSF-c neuron. Confocal image from a transverse spinal cord section with a CSF-c neuron (arrow) expressing somatostatin (green). A cilium (arrowhead) extending from the bulb-like ending is immunoreactive to α-tubulin (magenta). Scale bar, 10 µm.
